# Determining the Functional Oligomeric State of Membrane-Associated Protein Oligomers Forming Membrane Pores on Giant Lipid Vesicles

**DOI:** 10.1021/acs.analchem.2c05692

**Published:** 2023-05-06

**Authors:** Vandana Singh, Sabína Macharová, Petra Riegerová, Julia P. Steringer, Hans-Michael Müller, Fabio Lolicato, Walter Nickel, Martin Hof, Radek Šachl

**Affiliations:** † J. Heyrovský Institute of Physical Chemistry of the Czech Academy of Sciences, Dolejškova 3, 182 23 Prague, Czech Republic; ‡ Faculty of Mathematics and Physics, Charles University, Ke Karlovu, 2027/3, 121 16 Prague, Czech Republic; § Heidelberg University Biochemistry Center, Im Neuenheimer Feld 328, 69120 Heidelberg, Germany; ∥ Department of Physics, University of Helsinki, P.O. Box 64, FI-00014 Helsinki, Finland

## Abstract

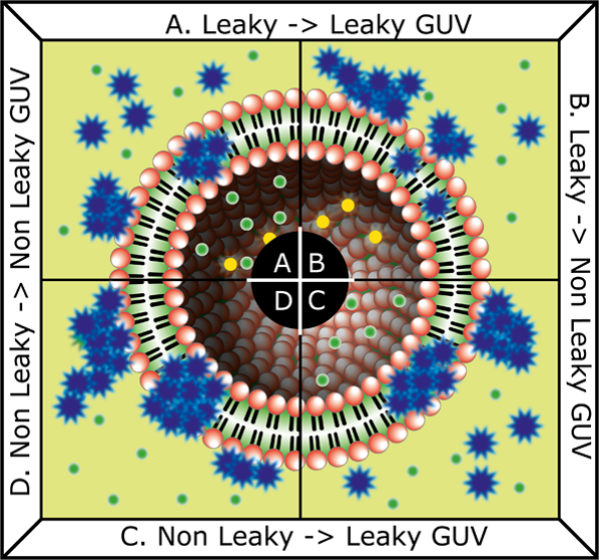

Several peripheral membrane proteins are known to form membrane pores through multimerization. In many cases, in biochemical reconstitution experiments, a complex distribution of oligomeric states has been observed that may, in part, be irrelevant to their physiological functions. This phenomenon makes it difficult to identify the functional oligomeric states of membrane lipid interacting proteins, for example, during the formation of transient membrane pores. Using fibroblast growth factor 2 (FGF2) as an example, we present a methodology applicable to giant lipid vesicles by which functional oligomers can be distinguished from nonspecifically aggregated proteins without functionality. Two distinct populations of fibroblast growth factor 2 were identified with (i) dimers to hexamers and (ii) a broad population of higher oligomeric states of membrane-associated FGF2 oligomers significantly distorting the original unfiltered histogram of all detectable oligomeric species of FGF2. The presented statistical approach is relevant for various techniques for characterizing membrane-dependent protein oligomerization.

## Introduction

A typical feature of many membrane-associated proteins is their oligomerization into functional units characterized by the oligomeric size *N* (m.u.), which is defined as the number of monomeric protein units (m.u.) in the cluster. Thanks to recent advances in high-resolution imaging techniques, the determination of the oligomerization states of membrane-associated proteins appears relatively straightforward.^1−5^ Frequently, recombinant proteins are reconstituted in model membrane systems comprising giant unilamellar vesicles (GUVs) or supported phospholipid bilayers (SPBs) and then exposed to examination using high-resolution microscopy.^6−15^ This offers many benefits, namely, having a clear-cut system with less complexity. The obtained distributions of oligomeric states, however, are often significantly multimodal and it is unclear if all of the oligomerization states identified belong to functional protein units or if they are merely the product of random protein aggregation.^6−15^ One example is the fibroblast growth factor 2 (FGF2) protein that oligomerizes at the membrane into multimers with a broad distribution of oligomer sizes.^16^ More specifically, stimulated emission depletion (STED) microscopy revealed dimers to 24-mers of FGF2-Halo-StarRed on SPBs.^16^ Similarly, brightness measurements based on fluorescence correlation spectroscopy (FCS) identified dimers up to 18-mers of FGF2-GFP using free-standing membranes of GUVs.^16,17^ Despite this apparent agreement, one shall consider potential problems connected with these systems: It has been shown that the support on which SPBs are prepared significantly slows down the diffusion of lipids and embedded proteins^15,18−20^ or even may immobilize proteins.^15^ Such impeded dynamics may imply that (1) protein oligomers that naturally form in cellular membranes will not form in model membrane systems or (2) some of the oligomers formed in these membranes will not be functional because of their nonspecific aggregation into nonfunctional multimeric units. In this regard, it appears crucial to distinguish between functional and nonfunctional protein oligomers that form in the in vitro model membrane systems where nonspecifically aggregated proteins may be present.

This fundamental problem inspired us to develop a single-molecule single-vesicle statistical approach called dual(+1)-FCS that enables to simultaneously measure the average protein oligomer size on a vesicle and confirm its functionality.^17^ In this work, we were able to significantly expand the potential of this technology by monitoring the oligomerization of FGF2 and the gradual permeabilization of the membrane that reports on protein insertion into the membrane over time. Thus, we repeatedly imaged the same group of GUVs to assess their oligomeric state and membrane permeabilization. This allowed us to observe how unspecific protein aggregation gradually increased the protein oligomer state. In fact, we could distinguish between two different protein populations: (1) unstable fraction of proteins assembled on intact vesicles without any apparent purpose: this nonnegligibly populated fraction of nonfunctional membrane-associated proteins exhibited a particular propensity to further assemble into bigger multimeric units with a variety of oligomeric states and (2) a fraction of stable membrane-inserted dimers to hexamers on permeabilized vesicles: by selecting from this heterogeneous ensemble of vesicles only permeabilized GUVs imaged shortly after the start of incubation, we were able to determine the functional oligomeric state of membrane-inserted protein oligomers forming membrane pores. Considering that in vivo only monomers up to trimers have so far been detected, this undistorted protein population of dimers to hexamers more closely mimics the distribution of FGF2 oligomers found in cellular plasma membranes.^21^

## Experimental Section

### Dual(+1)-FCS: Principle

Dual(+1)-FCS is a dual-color FCS (dual-FCS) assay^22^ with a third excitation–emission channel, hereafter called dual(+1)-FCS. The measurement is performed by placing the GUV membrane into the waist of 470 and 635 nm lasers and conducting 60-s-long dual-color FCS measurements. The autocorrelation (AC) FCS curves are fitted by a model assuming two-dimensional diffusion in the membrane
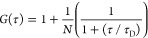
1where *N* is the number of diffusing molecules in the confocal volume, τ is a so-called lag time and τ_D,free_ the diffusion time of the diffusing fluorescent molecules (either FGF2-GFP in the blue channel or Abberior Star 635P dioleolyl phosphatidylethanolamine (DOPE) in the red channel). The blue fluorescence channel (excitation at 470 nm and emission at 505 ± 15 nm) is used to quantify the average protein surface concentration (PSC) and the average oligomeric state ⟨*N* (m.u.)⟩ of FGF2-GFP per GUV by a standard blue-color FCS.^23,24^ More specifically, as explained by Šachl et al.,^17^ this method gives access to the average fluorescence intensity of the oligomer ⟨*I*(oligo)⟩ and the average number of oligomers in the confocal spot ⟨*N*(oligo)⟩ (eq 1). The approach can thus be used to calculate the average brightness of an FGF2 oligomer per GUV as  and the average oligomeric state of FGF2 per vesicle by dividing ⟨ϕ(oligo)⟩ by the brightness of a monomer ⟨ϕ(mono)⟩ according to . In this calculation, it is assumed that the brightness of FGF2-GFP is directly proportional to *N* (m.u.) of FGF2-GFP,^21,25,26^ which, according to the existing literature, may introduce a slight offset in the determined oligomeric state.^27−29^ However, as shown in Figure SI1, by comparing the histograms of the oligomeric states for FGF2 carrying one of the two different fluorescent tags (either GFP or Halo-StarRed), this offset is below the resolution of this approach. Background correction was not performed as it had a negligible effect in our measurements. The approach further allows to calculate PSC as the number of protein molecules in the confocal spot *N*(oligo/mono) × *N* (m.u.) of a known beam waist radius ω: .^17^ The red channel (excitation at 630 nm and emission at 697 ± 29 nm) is used for visualization of all GUVs, correct vertical positioning of the membrane into the beam center, and quality check of the membrane by measuring the diffusion coefficient of Abberior Star 635P DOPE by red-color FCS and comparing this value to a priori known *D* of a fluorescently labeled lipid in the membrane. Finally, the green channel (excitation at 543 nm and emission at 590 ± 25 nm) is used to sort GUVs into leaky (permeabilized) and nonleaky (intact) GUVs by monitoring the passage of Alexa-Fluor-532 from the GUV exterior into the GUV interior.^17^ To determine whether the pores are permanently or only temporarily open, the fluorescent dye Alexa-Fluor-532 was added twice during the measurement: at time zero to reach the bulk concentration of 0.2 μM and then at time 180 min to reach the bulk concentration of 0.4 μM. Therefore, if the pores are permanently open, the dye concentration in the vesicle interior will match the dye concentration in the GUV exterior both after the first and second addition of the dye. On the other hand, if the pores are opened between the first and second measurement, the interior of the vesicles will initially be dark and fluorescent during the second measurement.

### Dual(+1)-FCS: Measurement

All measurements were performed on an Olympus FluoView 1000 MPE system upgraded with a dual detector channel PicoQuant laser scanning microscope (LSM) Upgrade Kit and a homebuilt excitation system consisting of LDH-D-C-470, LDH-D-C-640 diode laser heads, and 543 nm HeNe continuum wave lasers as previously described in Šachl et al.^17^ Briefly, individual GUVs were imaged using FluoView software and conventional FluoView 1000 Hardware and classified as leaky or nonleaky. The position coordinates of individual GUV were stored in memory, allowing for repeated dual(+1)-FCS measurements on a selected set of GUVs. After positioning the laser beam into the GUV membrane, the emission from the membrane was collected using HydraHarp400 Multichannel Picosecond Event Timer & time-correlated single photon counter (TCSPC) module, controlled via SymPhoTime64 software, which incorporates the control of the pulse diode laser (PDL) 828 Sepia II driver (PicoQuant, Berlin, Germany). The emission signal was correlated, and the obtained autocorrelation curves (AC) were fitted by a model assuming two-dimensional (2D) diffusion in the membrane and dye transition to the triplet state.^30^ In the final step, the obtained FCS output parameters were used to calculate the average PSC and *N* (m.u.) of FGF2 on each selected GUV, as well as *D*(Abberior Star 635P DOPE).

### GUV Preparation

GUVs mimicking the composition of plasma membranes (33 mol % bovine liver extracted PC (phosphatidylcholine), 9 mol % bovine liver extracted PE (phosphatidylethanolamine), 5 mol % porcine brain extracted PS (phosphatidylserine), 5 mol % bovine liver extracted PI (phosphatidylinositol), 15 mol % chicken egg extracted SM (sphingomyelin), 30 mol % ovine extracted cholesterol, 1 mol % 1,2-dioleoyl-*sn*-glycero-3-phosphoethanolamine-*N*-(biotinyl) (sodium salt) Biotinyl-PE, 2 mol % porcine brain PI(4,5)P_2_ (phosphatidylinositol-4,5-bisphosphate), and 0.05 mol % Abberior Star 635P DOPE fluorescent probe) were prepared by the electroswelling method.^31^ More specifically, 1.5 mM lipid mixture (in chloroform) was deposited on platinum electrodes, and the remaining organic solvent was dried by evaporation. The lipid film coated electrodes were inserted into a titanium chamber filled with 300 mM sucrose buffer (300 mOsm/kg), and electroswelling was performed at 45 °C at an alternating electric field of 10 Hz and peak-to-peak voltage of 4 V for 50 min and 2 Hz and 4 V for 20 min. To replace the external buffer, the GUVs were washed with an excess of HEPES buffer (25 mM HEPES; 150 mM NaCl; pH 7.4; 305 mOsm/kg) in two rounds of centrifugation (1200*g* at 25 °C for 5 min). The supernatant was removed, and the remaining pellet was resuspended in 400 μL of HEPES buffer. To immobilize the GUVs on the surface of Ibidi uncoated imaging chambers, the surface was coated with 0.1 mg/mL Biotin-BSA (Sigma-Aldrich) and 0.1 mg/mL Neutravidin (Thermo fisher scientific) prior to addition of Biotinyl-PE containing GUVs (for the precise composition of GUVs, see the beginning of this section). For Dual(+1)-FCS measurements, an imaging chamber contained 200 nM FGF2-GFP, resuspended GUVs, and 200 nM Alexa-Fluor-532 in the final volume of 350 μL of HEPES buffer. All lipids were purchased from Avanti Polar Lipids.

### Protein Expression and Purification

His-tagged variants of FGF2-GFP (pET15b) were expressed in *Escherichia coli* strain BL21 Star (DE3). All proteins were purified in three steps via Ni-NTA affinity chromatography, heparin chromatography, and size-exclusion chromatography using a Superdex 75 column.

## Results and Discussion

### Distinguishing Specific from Unspecific Fibroblast Growth Factor 2 in-Membrane Oligomerization by Dual(+1)FCS

As STED microscopy and FCS on SPBs and GUVs, respectively, showed broad size distributions of membrane-bound FGF2 oligomers, we were asking whether this might not be the consequence of capturing both specific and unspecific protein–protein interactions.^16^ FGF2 performs most of its important functions in the extracellular environment, where it is translocated across the plasma membrane using the type I unconventional secretion pathway.^32,33^ In an in vitro reconstituted system, the most important steps of this process, listed in chronological order, include (1) binding to PI(4,5)P_2_;^34,35^ (2) in-membrane oligomerization;^36^ (3) insertion of the protein into the membrane that is accompanied by membrane permeabilization; and (4) heparan sulfate-assisted release of FGF2 into the luminal buffer^16^ mimicking cell surface heparan sulfates.^37−39^ Based on step 3 of this mechanism, we suggest using the permeability of the membrane as an indicator for membrane insertion of FGF2. This suggestion finds support by experiments that showed that upon membrane insertion of FGF2, membrane passage of small fluorescent tracers and trans-bilayer diffusion of lipids are observed simultaneously.^36^

To distinguish between specific and unspecific FGF2 in-membrane protein–protein interactions, we here employ a recently developed dual(+1)-FCS. Dual(+1)-FCS approach is a dual-color FCS where an additional third excitation–emission channel is used to detect membrane permeabilization (pore formation) by the influx of a green fluorescent leakage dye into the GUV interior.^17^ The FCS measurement is performed on a large set of leaky and nonleaky GUVs in the upper part of the GUV membrane: the blue (protein) emission channel serves to get information about the size of membrane-associated protein oligomers and membrane surface concentration of the protein, whereas the red (lipid) emission channel is used to place the membrane into the beam center and quality-check the membrane.^17^ In this way, membrane permeabilization can be correlated with the readouts of FCS: molecular brightness of GFP, which is directly proportional to the oligomer size,^21,25,26^ protein surface concentration, and the diffusion coefficient of the protein oligomer. Since membrane permeabilization indicates the insertion of FGF2 into the membrane, one can divide the observed GUVs into two distinct categories: leaky GUVs containing membrane-inserted FGF2 and nonleaky GUVs where protein insertion is questionable.

### Statistical and Time-Dependent Analysis of Dual(+1)-FCS Experiments: Definition of INITIAL and FINAL States

**Figure 1:**
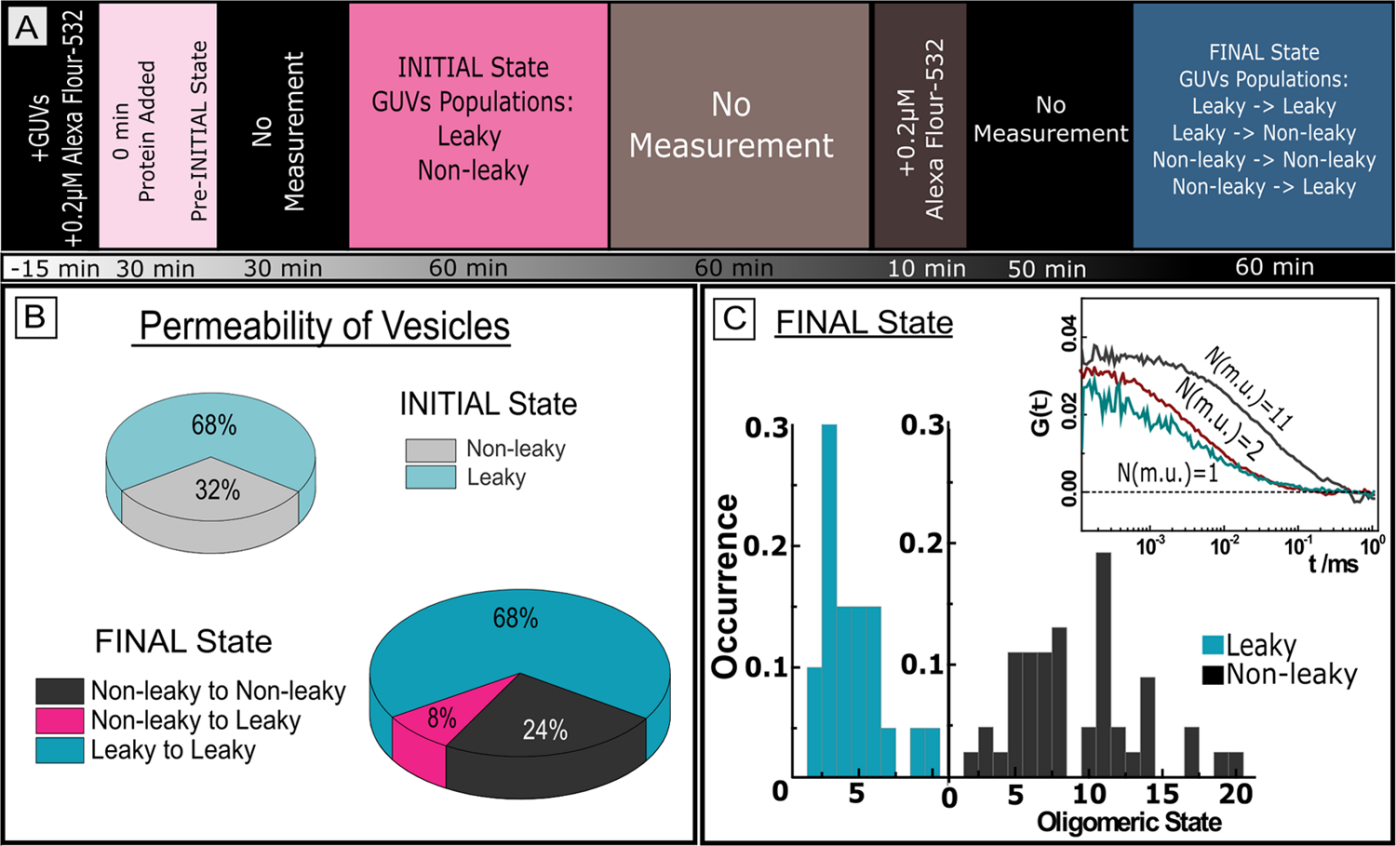
Time-dependent analysis of the dual(+1)-FCS experiments. (A) Timeline of a dual(+1)-FCS experiment: At time *t* = −15 min, GUVs together with Alexa-Fluor-532 dissolved at the bulk concentration of 0.2 μM were added into the imaging chamber. This step was followed by adding FGF2-GFP to the chamber at *t* = 0 min and incubation of the protein with the GUVs for 60 min. Any measurement performed between *t* = 0 min and *t* = 30 min characterizes a so-called pre-INITIAL state. The INITIAL state corresponds to the measurements made between *t* = 60 min and *t* = 120 min. As shown in the upper pie graph in panel (B), 68 ± 3% of GUVs have already been leaky at that time. The FINAL state characterizes the GUV systems that are under equilibrium (*t* ≥ 240 min). In order to find out whether the given GUVs were constantly permeabilized, at time *t* = 180 min, an additional amount of the fluorescent tracer was added (see also Experimental Section). In this FINAL equilibrium state, 24 ± 1% of all GUVs remained intact, representing the population of (nonleaky → nonleaky) GUVs (lower pie graph in panel B). The fraction of leaky vesicles increased about 8 ± 1% and represents the population of (nonleaky → leaky) GUVs. A control sample containing no protein exhibited a leakage of 12 ± 1% in the INITIAL and 14 ± 2% in the FINAL state. (C) Histogram of oligomeric states of FGF2-GFP on permeabilized (blue columns) and intact GUVs (black columns) in the FINAL state. The inset shows representative autocorrelation curves for ⟨*N* (m.u.)⟩ = 1, ⟨*N* (m.u.)⟩ = 2, and ⟨*N* (m.u.)⟩ = 11.

From the experiment, to obtain an approximate estimate of the time scale on which protein oligomerization occurs, we set up the dual(+1)-FCS experiment in the following way (see also Figure 1A): prior to the start of the experiment, GUVs dissolved in a buffer containing the leakage tracer Alexa-Fluor-532 were immobilized on the surface of an imaging chamber. The experiment started by adding the protein into the chamber and incubation of the GUVs with FGF2 for at least 60 min. The dual(+1)-FCS measurement carried out between 60 and 120 min after the start of the experiment defines a so-called INITIAL state. At the time of 60 min after the start of the experiment, 68 ± 3% of all GUVs have already leaked, and 32 ± 2% of the GUVs still have an intact membrane (Figure 1B). At time *t* = 180 min, a further dose of the fluorescent tracer was administered to determine whether the observed GUVs were continuously permeable. Importantly, the percentage of leaky vesicles only slightly increased to 76 ± 4% during the course of the following 180 min, while the rest stayed intact. At this point, only 14 ± 2% of all GUVs are leaky in the absence of FGF2. We assume that the second measurement performed no earlier than 240 min after the start of the experiment describes the FINAL equilibrium state.

### Analysis of the FINAL State

In our analysis, we first set out to characterize the FINAL state in which the system appears to be under equilibrium. In total, we characterized 67 GUVs based on their permeability and determined ⟨*N* (m.u.)⟩ as well as PSC of all of these GUVs. By histograming ⟨*N* (m.u.)⟩, determined separately on leaky and nonleaky GUVs, we identified two clearly distinct populations of GUVs differing in the average oligomer size of FGF2 (⟨*N* (m.u.)⟩). A relatively narrow population of permeabilized GUVs contains membrane-inserted FGF2 where dimers to hexamers represent the main oligomer species. In contrast, an ensemble of intact GUVs has a broad distribution of oligomer sizes where protein insertion is questionable (Figure 1C). This latter population is represented by significantly larger protein aggregates, mainly hexamers, and 12-mers.

**Figure 2:**
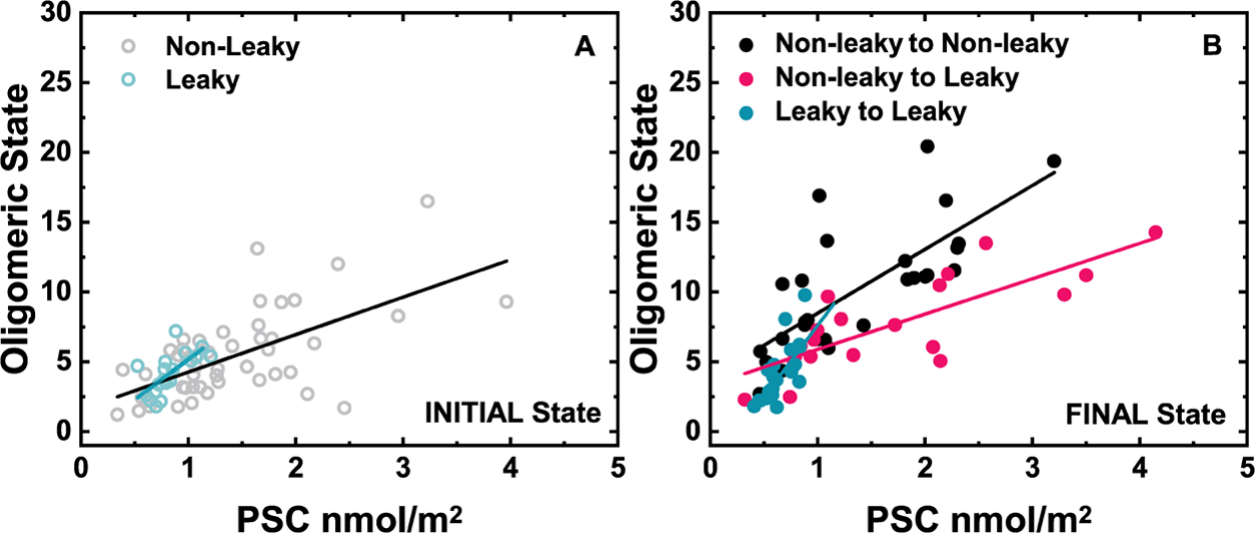
FGF2 oligomeric-state characterization on individual GUVs. Correlation of the average oligomeric state and protein surface concentration of FGF2-GFP for the INITIAL (A) and FINAL (B) states. Each point in the graph corresponds to data obtained on one vesicle. Depending on the membrane permeability of each GUV in both the INITIAL and FINAL states, the GUVs were divided into four different categories: (leaky → leaky), (nonleaky → nonleaky), (nonleaky → leaky), and (leaky → nonleaky) GUVs. (Leaky → nonleaky) vesicles are not shown in the graph as GUVs with such history, with rare exceptions, do not exist. The solid lines represent the fits to the data.

For the purpose of more detailed characterization of FGF2 on individual GUVs, we constructed a 2D scatter plot that relates the average oligomer size and protein surface concentration of FGF2 for each GUV (Figure 2). We further divided the individual GUVs not only based on their permeability in the FINAL state but also according to whether the given GUV was leaky or intact in the INITIAL state. In principle, there are four different GUV categories: (1) GUVs with permanently open pores between the first and second measurements (leaky → leaky); (2) GUVs with an impermeable membrane in both the INITIAL and FINAL states (nonleaky → nonleaky); (3) GUVs that become leaky after the first measurement is performed (nonleaky → leaky); and (4) GUVs where pores close after the initial measurement (leaky → nonleaky). Since we could find only 1 GUV belonging to the fourth population, we do not discuss this population further. The absence of the fourth population is probably caused by the lack of heparan sulfates in the model GUV system. These molecules are required to complete FGF2 translocation by disassembling FGF2 oligomers in cells and closing the pores.^38−41^

Interestingly, there is a significant difference between the 2D scatter plot characterizing the FINAL state for (leaky → leaky) GUVs and the plot for either (nonleaky → nonleaky) or (nonleaky → leaky) GUVs: whereas the data points for (leaky → leaky) GUVs are localized exclusively at the PSC(FGF2-GFP) of less than 1 nmol/m^2^ and at ⟨*N* (m.u.)⟩ of less than 10 with the average oligomer size ⟨*N* (m.u.)⟩ = 4.21 ± 2.073 and PSC = 0.64 ± 0.139 nmol/m^2^, the data points for (nonleaky → nonleaky) and (nonleaky → leaky) GUVs are significantly more scattered over a broad range of PSC ∈ ⟨0;4.1⟩ as well as ⟨*N* (m.u.)⟩ ∈ ⟨1;20⟩ and shifted toward higher PSC and ⟨*N* (m.u.)⟩. Whereas in the case of (nonleaky → nonleaky) GUVs, ⟨*N* (m.u.)⟩ = 10.3 ± 4.439 and PSC = 1.39 ± 0.716 nmol/m^2^, and in the case of (nonleaky → leaky) GUVs, ⟨*N* (m.u.)⟩ = 7.64 ± 3.256 and PSC = 1.7 ± 1.23 nmol/m^2^. Moreover, the oligomer size increases with the increasing protein surface concentration. However, this dependence appears much steeper for (leaky → leaky) GUVs in comparison to the other two sets (Figure 2B).

These results thus indicate that the mechanism of FGF2 oligomerization on disrupted and intact GUVs is different. On (leaky → leaky) GUVs, a considerable fraction of FGF2 is membrane-inserted, which allows for specific oligomerization of the protein by means of cysteine C95 and C77.^16^ This hypothesis is further supported by the aforementioned steep dependence of ⟨*N* (m.u.)⟩ on PSC on (leaky → leaky) GUVs, being an expected consequence of increased sensitivity of ⟨*N* (m.u.)⟩ to PSC in the case where oligomerization is driven specifically. The opposites of this population of GUVs are (nonleaky → nonleaky) vesicles, on which protein insertion is highly controversial. Here, nonspecific oligomerization of FGF2 results in the formation of noticeably larger aggregates, the self-assembly of which requires higher PSC. Since this population of oligomers lacks the specificity that drives protein oligomerization, the resulting dependence of ⟨*N* (m.u.)⟩ on PSC appears less steep and more chaotic with a less obvious trend (Figure 2). The remaining population of (nonleaky → leaky) GUVs displayed in Figure 2 becomes leaky typically with a lag time of 60–180 min. This population of GUVs must also contain the inserted protein in the FINAL state (notice that the membrane of these GUVs is permeabilized in the FINAL state), but its presence is overshadowed by nonspecifically aggregated protein oligomers that formed in excess during the INITIAL state. For this reason, this population of vesicles has properties more similar to (nonleaky → nonleaky) GUVs containing nonfunctional aggregated proteins in excess.

### Analysis of the INITIAL State

**Figure 3:**
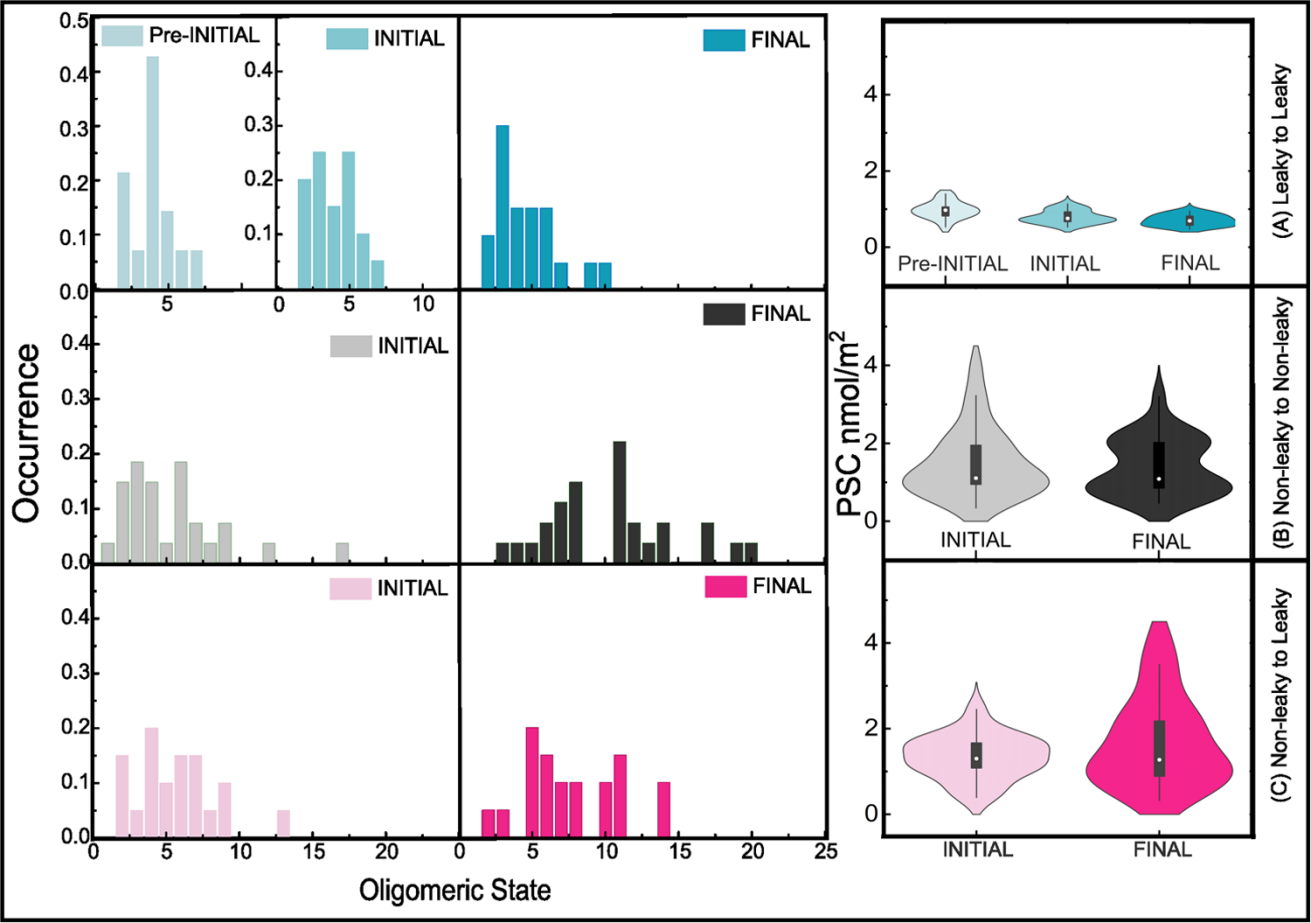
Time-dependent FGF2 oligomer size distribution. The distribution of protein oligomer sizes and protein surface concentrations are shown for the INITIAL and FINAL states and the pre-INITIAL state in the case of (leaky → leaky) GUVs. The histograms were constructed for three distinct GUV categories: (A) (leaky → leaky, upper row), (B) (nonleaky → nonleaky, middle row), and (C) (nonleaky → leaky, lower row) GUVs.

Since the fraction of nonspecifically aggregated proteins may increase over time, we also characterized the resulting distribution of oligomer sizes in the INITIAL state, i.e., 180 min earlier. First, we examined the INITIAL state of (leaky → leaky) GUVs on which specifically self-assembled FGF2 oligomers are in excess. Interestingly, dimers to hexamers represent the most dominant species on leaky GUVs, already in the INITIAL state (Figure 3A). Since the histograms constructed for the INITIAL and FINAL (Figure 3A) states on those GUVs look similar, we first concluded that membrane-inserted protein oligomers do not aggregate over time and second that specific oligomerization of FGF2 takes place at the time scale shorter than 60 min. This result agrees with recently published single-molecule cell experiments that showed that translocation of FGF2 across the plasma membrane occurs in the order of hundreds of milliseconds.^21^ A closer look at Figure 3A, however, reveals a small fraction of large oligomers in the FINAL state on (leaky → leaky) GUVs that is completely absent in the INITIAL state. It is, therefore, more accurate to consider only the histogram obtained for the INITIAL state when characterizing the population of functional membrane-inserted FGF2.

The situation is significantly different for the population of (nonleaky → nonleaky) and (nonleaky → leaky) GUVs on which FGF2 aggregates nonspecifically. Here, large protein clusters present in the FINAL state are largely absent in the INITIAL state. In this state, dimers to hexamers represent the most dominant oligomer species not only on leaky but also on nonleaky GUVs (Figure 3). Overall, it follows from the results presented so far that in order to determine the unbiased oligomeric state of membrane-inserted FGF2 that is unaffected by apparently present nonfunctional aggregated proteins, GUVs need to be categorized, and the most accurate information about functional membrane-inserted FGF2 oligomers is provided by the population of leaky GUVs in the INITIAL state.

### Is There a Pre-INITIAL State?

Considering the disproportionality between the short time of FGF2 translocation in cells^21^ and the 60-min-long incubation time that was used in the experiment, we decided in the next step to reduce the incubation time as much as possible. Therefore, we started the first measurement immediately after adding the protein to the vesicles and continued to measure additional GUVs for 30 min. For simplicity, we focused only on vesicles that were leaky from the very beginning. We then compared the received histograms of protein oligomeric states (corresponding to the so-called pre-INITIAL state according to our definition), with the histograms for the INITIAL state (Figure 3A). A comparison of these histograms shows that the given distributions do not evolve over time. Thus, the measurement carried out in the INITIAL state provides objective information about the distribution of membrane-inserted FGF2 oligomers on the membrane. It also shows that the formation of specifically self-assembled FGF2 oligomers is beyond the resolution of this approach.

### Correlated Changes between the INITIAL and FINAL States

**Figure 4:**
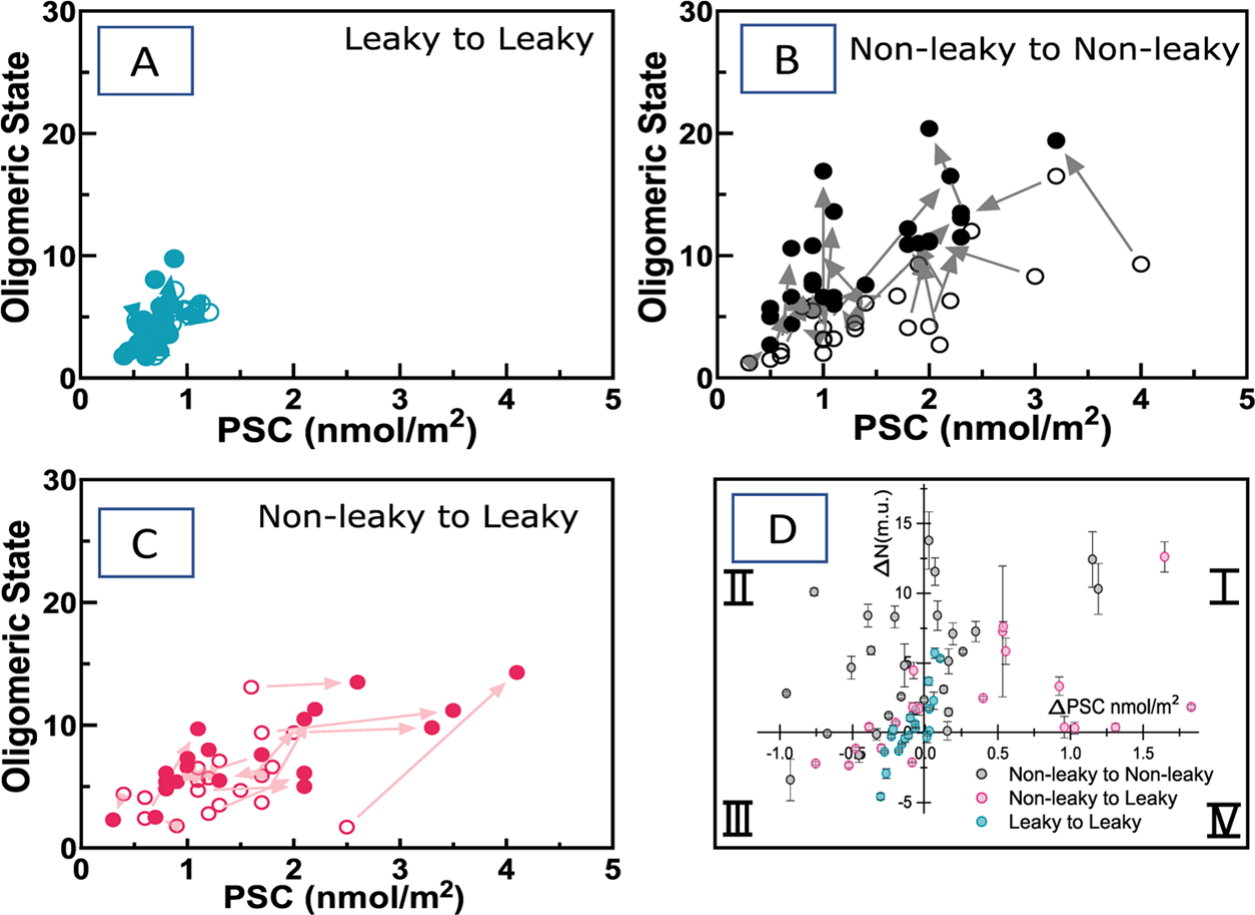
Time-tracking oligomeric state and protein surface concentration transitions. (A–C) Arrow plots indicate both the oligomeric state and protein surface concentration on individual GUVs for the INITIAL and FINAL states. Each pair of dots in the diagram corresponds to a single GUV. The arrow plots are shown for (A) (leaky → leaky), (B) (nonleaky → nonleaky), and (C) (nonleaky → leaky) GUVs. In panel (D), the changes, Δ, in ⟨*N* (m.u.)⟩ and PSC are correlated against each other.

Since dual(+1)-FCS enables the measurement of the oligomer size and PSC repeatedly on the same GUV, individual vesicles can be tracked in time. In this way, ⟨*N* (m.u.)⟩ and PSC determined in the INITIAL state can be correlated with ⟨*N* (m.u.)⟩ and PSC on the same GUV in the FINAL state. To demonstrate the applicability of this approach, we took the data set shown in the 2D scatter plots in Figure 2 recorded on the same set of GUVs in both the INITIAL and FINAL states and replotted them in the form of 2D “arrow” plots (Figure 4A–C) as well as 2D “delta” plots (Figure 4D). This allowed for ⟨*N* (m.u.)⟩ and PSC measured in both states as well as changes in *N* (m.u.) and PSC, i.e., Δ*N* (m.u.) and ΔPSC, to be correlated against each other. In Figure 4A–C, we compare a 2D arrow plot for (leaky → leaky) GUVs with the ones obtained for (nonleaky → nonleaky) and (nonleaky → leaky) GUVs. At first glance, the two sets of arrow plots differ from each other. Whereas the transitions between the INITIAL and FINAL states on (leaky → leaky) GUVs exhibit low variability and occur exclusively at low PSC, the transitions on (nonleaky → nonleaky)/(nonleaky → leaky) GUVs are more scattered and accompanied by a large increase in the oligomer size and PSC. For a more detailed quantification of the changes that occurred, we further focus on the analysis of the displayed delta plot (Figure 4D). This allows the (leaky → leaky), (nonleaky → leaky), and (nonleaky → nonleaky) vesicle populations to be further divided into four adjacent quadrants according to whether there was a simultaneous increase in ΔPSC and Δ*N* (m.u.) (quadrant I), a decrease in ΔPSC and increase in Δ*N* (m.u.) (quadrant II), a decrease in both ΔPSC and Δ*N* (m.u.) (quadrant III), or an increase in ΔPSC and decrease in Δ*N* (m.u.) (quadrant IV). Even in this graph, the (leaky → leaky) vesicle population looks noticeably different from the other two populations. Events are more or less evenly distributed among quadrants I to III, with roughly the same number of vesicles showing either positive or negative Δ*N* (m.u.) or ΔPSC values (Table 1). Furthermore, while the changes in PSC are small, reaching a relative change of only about 10–20%, the changes in ⟨*N* (m.u.)⟩ reach the maximum of +3 monomeric units in quadrant I and only +0.8 m.u. in quadrant II. In contrast, in the case of (nonleaky → nonleaky) vesicles, 85% of all GUVs show an increase in ⟨*N* (m.u.)⟩, with Δ*N* (m.u.) in the first quadrant reaching up to seven monomer units on average. In the (nonleaky → leaky) population containing a fraction of specifically oligomerized FGF2, this change is less pronounced but still large: 75% of all vesicles show an increase in ⟨*N* (m.u.)⟩, with +4 monomer units in quadrant I. As for the evolution in PSC, roughly the same fractions of vesicles show a decrease or increase in PSC in (nonleaky → nonleaky) or (nonleaky → leaky) GUV populations. Furthermore, the ΔPSC in quadrant I is up to 19 times larger on this population of vesicles in comparison to (leaky → leaky) vesicles on which nonspecific protein aggregation is insignificant (see again Table 1). Overall, these results support our theory that specific oligomerization takes place preferentially on (leaky → leaky) vesicles. In this case, it is anticipated that the transitions between the INITIAL and FINAL states will be more defined; in contrast to nonspecific oligomerization on intact vesicles, with more pronounced and scattered transitions subject to no rules.

**Table 1:** Changes in Both the Average Protein Surface Concentration and Average Protein Oligomeric States Calculated for Quadrants I–IV as Well as for All GUV Populations under Consideration^a^

		nonleaky to nonleaky			nonleaky to leaky			leaky to leaky		
quadrant	[Δ*N* (m.u.), ΔPSC]	no. of GUVs	⟨Δ*N* (m.u.)⟩	⟨ΔPSC⟩	no. of GUVs	⟨Δ*N* (m.u.)⟩	⟨ΔPSC⟩	no. of GUVs	⟨Δ*N* (m.u.)⟩	⟨ΔPSC⟩
I	[+,+]	12	7.2 ± 4.34	0.32 ± 0.408	10	4.2 ± 4.04	0.98 ± 0.50	6	3.3 ± 2.16	0.05 ± 0.030
II	[+,−]	11	4.8 ± 3.04	–0.35 ± 0.295	5	1.8 ± 1.61	–0.14 ± 0.13	5	0.8 ± 0.52	–0.11 ± 0.072
III	[−,−]	4	–1.3 ± 1.56	–0.60 ± 0.254	5	–1.8 ± 0.59	–0.42 ± 0.246	7	–1.3 ±1.57	–0.21 ± 0.071
IV	[−,+]							2	–0.2 ± 0.19	0.01 ± 0.003

a Comparing previous results on the oligomeric state of FGF2 on GUVs, SPBs, and cells with the present statistical and time-dependent analysis reveals relevant FGF2-GFP membrane oligomers to range between dimers and hexamers.

Oligomerization and insertion of FGF2 into the membrane are necessary prerequisites for the successful translocation of the protein across the membrane. The first evidence for PI(4,5)P_2_-dependent oligomerization of FGF2 was provided by FGF2-liposome binding experiments analyzed by reducing sodium dodecyl sulfate-polyacrylamide gel electrophoresis (SDS-PAGE) followed by Western Blot^36^ as well as nonreducing SDS- and native PAGE that identified dimers and higher oligomers associated with the membrane.^37^ These relatively invasive experiments were later supported by fluorescence cross-correlation experiments that detected the codiffusion of FGF2-Atto488 with FGF2-Atto655 as well as codiffusion of phosphorylated variants (FGF2-Y81pCMF) of these proteins in GUVs.^36^ In 2017, we published the first size distributions of FGF2-Y81pCMF-Halo-StarRed oligomers in supported phospholipid bilayers using STED microscopy and of FGF2-Y81pCMF-GFP in GUVs by brightness-FCS analysis.^16^ In this work, STED revealed a complex size distribution of FGF2-Y81pCMF-Halo-StarRed oligomers with major components being represented by 3, 7, 11, and 17 monomers per cluster. Interestingly, we obtained strikingly similar size distribution of nonphosphorylated FGF2-GFP in the current work if the individual GUVs have not been sorted in any way, i.e., if all vesicles were taken into the analysis, regardless of whether they contained specifically or nonspecifically self-assembled oligomers (Figure SI1). In the same work by Steringer et al.,^16^ we attempted to determine the oligomerization states of FGF2-Y81pCMF-GFP separately on intact and pore-containing membranes. In the context of the current work, the conditions of this measurement resembled the measurement of the FINAL state. That was the first time we could detect noticeable differences in the oligomerization behavior on permeabilized and intact GUVs. We further modified the developed assay in such a way that it allowed repeated measurements on the same group of vesicles, whereby changes in membrane permeability could be directly correlated to potential changes in the clustering of membrane-associated proteins.^17^ Since membrane permeabilization is indicative of FGF2 oligomerization and membrane insertion, we could use this assay to discriminate functional from nonfunctional protein oligomers.

In the current work, we have expanded the potential of dual(+1)-FCS by monitoring oligomerization of FGF2 in a time-dependent manner and on statistically significant set of GUVs. We thus used dual(+1)-FCS to measure the oligomeric state and PSC of FGF2 and membrane permeability on the same group of GUVs three times in a row, shortly after starting the incubation of the protein with the GUVs, 60 or 240 min later. In this way, we were able to identify three diametrically opposed populations that would have been unidentifiable without the thorough filtering presented here (Figure 3): (1) a fraction of membrane-inserted dimers to hexamers on the vesicles with permanently open pores that were stable and almost no further oligomerized; (2) a fraction of membrane-associated nonspecifically aggregated proteins on permanently intact vesicles; more precisely dimers to 10-mers, which tended to aggregate into even larger aggregates over time and whose insertion in the membrane was controversial; and (3) a portion of predominantly aggregated proteins on the vesicles that started to leak usually after a delay of between 60 and 180 min. Although, in the FINAL state, this population of GUVs also contains a fraction of inserted proteins, its abundance is insignificant, making the resulting distribution of oligomeric states more similar to population 2. Thus, by analyzing protein oligomerization states exclusively on permeabilized GUVs imaged shortly after the start of incubation, we could narrow down the initially wide size distribution of FGF2 oligomers, which were observed in previous studies and ranged from monomers to 20-mers, to only dimers to hexamers. This “unbiased” population represents in our experiments membrane-embedded FGF2 oligomers. Considering the fact that catching the genuine translocating intermediates in cellular membranes is difficult due to relatively infrequent translocation events, this finding comes near to the distribution of FGF2 oligomers reported in cellular plasma membranes in which dimers and trimers represented the most dominant species.^21,36^

## Conclusions

In conclusion, here, we present robust and simple technology that can effectively discriminate between functional and nonfunctional membrane-associated oligomers. Using the example of fibroblast growth factor 2, we illustrated that broad distributions of protein in-membrane oligomer states can be a consequence of the existence of a mixture of complexes with different properties. By expanding the potential of dual(+1)-FCS with time-dependent measurements, we could monitor oligomerization of membrane-associated FGF2 proteins into functional and nonfunctional aggregates over time. In the specific case of FGF2, we were able to observe a gradual increase in the protein oligomer state caused by unspecific protein aggregation. More specifically, the approach revealed two distinct populations of FGF2: a population represented mainly by dimers to hexamers sharing properties with FGF2 oligomers detected in the plasma membrane of cells, and a population of nonspecifically aggregated proteins. Caution is thus required when constructing histograms of oligomeric states, as not all captured oligomers must necessarily be the result of specific oligomerization.

## Supporting Information Available

Distributions of oligomeric states for FGF2-GFP and FGF2-Y81pCMF-Halo-StarRed (Figure SI1); oligomeric state vs diffusion coefficient vs. protein surface concentration of FGF2 (Figure SI2); and output parameters from the dual(+1)-FCS method for all vesicles analyzed in this work (Table SI1) (PDF)
