# Supplementary material for: Determining the Functional Oligomeric State of Membrane-Associated Protein Oligomers Forming Membrane Pores on Giant Lipid Vesicles
Source: Anal Chem. 2023 May 6;95(23):8807–15. doi: 10.1021/acs.analchem.2c05692 (PMC10267887; doi:10.1021/acs.analchem.2c05692)
Supplement: Supplementary file 1 — ac2c05692_si_001.pdf [file ac2c05692_si_001.pdf]

Determining the functional oligomeric state of membrane-associated protein oligomers forming membrane pores on giant lipid vesicles

SUPPLEMENTARY INFORMATION

Vandana Singh[1,2], Sabína Macharová[1,#], Petra Riegerová[1,#], Julia P. Steringer[3], Hans-Michael Müller[3], Fabio Lolicato[3,4], Walter Nickel[3], Martin Hof[1], Radek Šachl[1,*]

[1]J. Heyrovský Institute of Physical Chemistry of the Czech Academy of Sciences, Dolejškova 3 182 23 Prague, Czech Republic;

[2] Faculty of Mathematics and Physics, Charles University, Ke Karlovu, 2027/3, 121 16 Prague, Czech Republic

[3]Heidelberg University Biochemistry Center, Im Neuenheimer Feld 328, 69120 Heidelberg, Germany

[4]Department of Physics, University of Helsinki, P.O. Box 64, FI-00014 Helsinki, Finland

[#]Contributed equally

[*]Correspondence:

Radek Šachl
radek.sachl@jh-inst.cas.cz

Table of contents

Figure SI1: Distributions of oligomeric states for FGF2-GFP and for FGF2-Y81pCMF-Halo-StarRed **S2**

Figure SI2: Oligomeric state vs. diffusion coefficient vs. protein surface concentration of FGF2 **S3**

Table SI1: Output parameters from the dual(+1)-FCS method for all vesicles analyzed in this work

S4

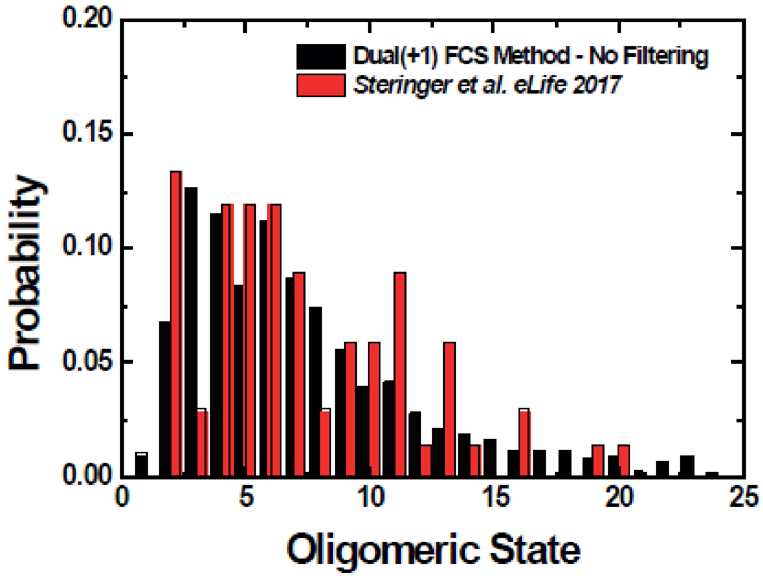

Figure SI1: Distributions of oligomeric states similar in shape measured 1) for FGF2-GFP in this work without using any filtering (i.e. without classifying GUVs on leaky and non-leaky ones) and 2) for FGF2-Y81pCMF-Halo-StarRed in the previous work by Steringer et al., eLife 2017[1].

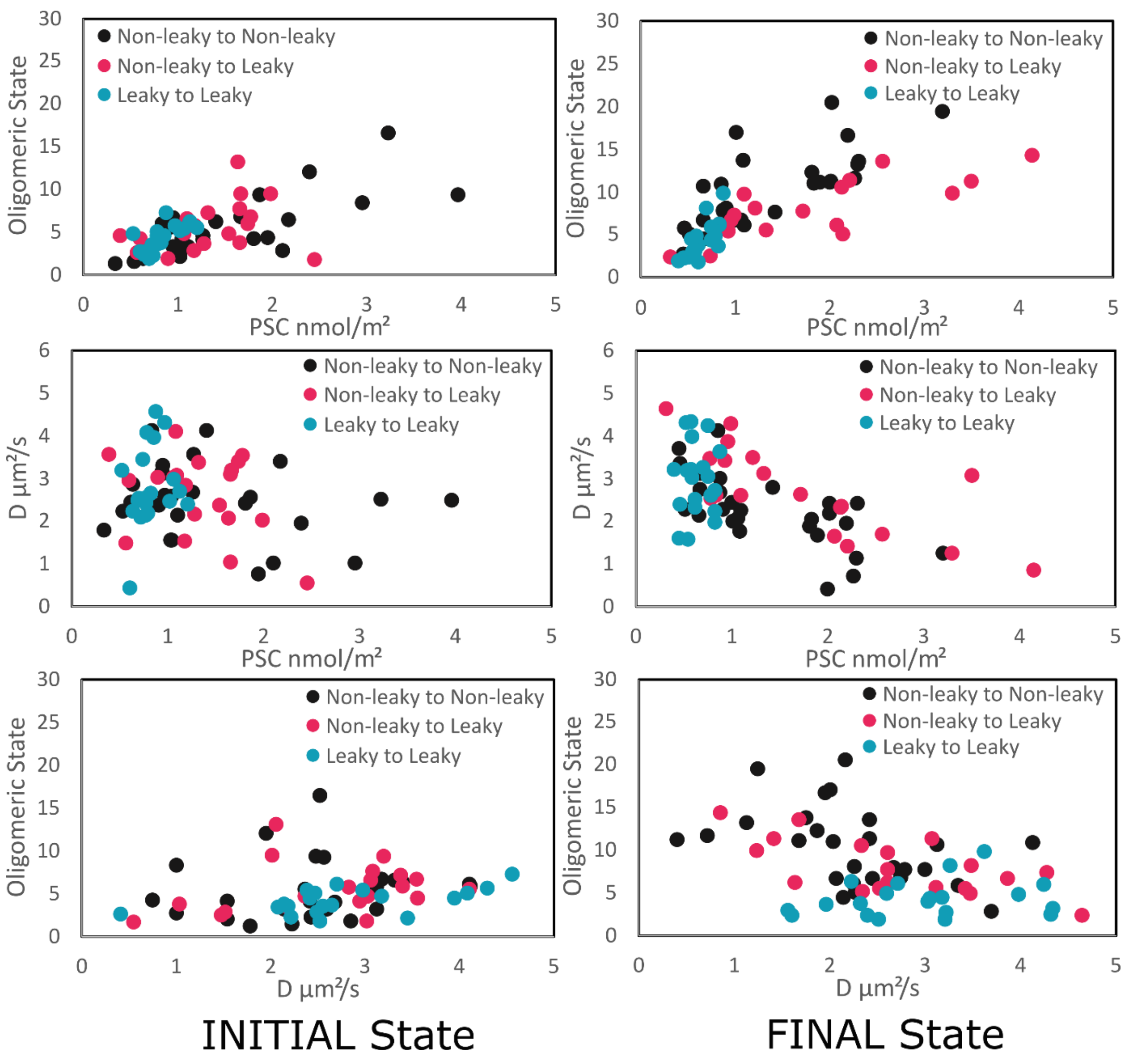

Figure SI2: Correlation of the average oligomeric state to PSC (upper row), average diffusion coefficient to PSC (middle row), as well as the oligomeric state to the diffusion coefficient of FGF2 (lower row) for all three populations considered throughout the work.

Table SI1: Output parameters from the dual(+1)-FCS method for all vesicles analyzed in this work. The parameters listed in Table 1 are: the average number of diffusing oligomers in the confocal volume ($\langle N \rangle$), the average fluorescence intensity from the confocal spot, the oligomer brightness ($\langle \phi \rangle$), the average number of monomer units per oligomer ($\langle N(\mathrm{m.u.}) \rangle$), the average protein surface concentration in the membrane (PSC) and the average diffusion coefficient of the oligomer ($\langle D \rangle$).

Vesicles are divided into three populations:

A: (Leaky → leaky) GUVs

			INITIAL state						FINAL state					
	day	GUV	$\langle N \rangle$	average intensity ⟨cps⟩	$\langle \phi \rangle$	$\langle N(\mathrm{m.u.}) \rangle$	PSC* nmol/m²	$\langle D \rangle$ µm²/s	$\langle N \rangle$	average intensity ⟨cps⟩	$\langle \phi \rangle$	$\langle N(\mathrm{m.u.}) \rangle$	PSC* nmol/m²	$\langle D \rangle$ µm²/s
1	8	B	18.0 ± 0.00	9.60E+03	0.35 ± 0.00	2.4 ± 0.00	0.6	0.42 ± 0.0	17.0 ± 0.0	8.25E+03	0.39 ± 0.00	2.7 ± 0.00	0.6	4.32 ± 0.0
2	8	C	16.6 ± 1.80	1.15E+04	0.46 ± 0.06	3.2 ± 0.44	0.7	2.08 ± 0.50	15.8 ± 1.80	7.19E+03	0.37 ± 0.07	2.6 ± 0.48	0.5	1.61 ± 0.50
3	8	D	30.2 ± 1.68	1.11E+04	0.24 ± 0.02	1.7 ± 0.13	0.7	2.52 ± 1.53	9.6 ± 1.68	9.35E+03	0.79 ± 0.06	5.5 ± 0.41	0.7	3.98 ± 1.53
4	8	E	19.1 ± 3.82	1.09E+04	0.38 ± 0.09	2.6 ± 0.61	0.6	2.49 ± 0.62	11.6 ± 3.82	9.30E+03	0.65 ± 0.07	4.5 ± 0.50	0.7	3.04 ± 0.62
5	8	F	15.5 ± 1.81	1.69E+04	0.72 ± 0.08	5.0 ± 0.55	1.0	2.98 ± 0.42	13.6 ± 1.81	1.19E+04	0.71 ± 0.04	4.9 ± 0.28	0.9	3.06 ± 0.42
6	8	G	17.3 ± 0.79	1.91E+04	0.73 ± 0.04	5.1 ± 0.32	1.1	2.39 ± 0.54	18.0 ± 0.79	1.31E+04	0.59 ± 0.03	4.1 ± 0.25	1.0	1.96 ± 0.54
7	8	H	17.7 ± 2.16	1.31E+04	0.49 ± 0.07	3.4 ± 0.52	0.8	2.65 ± 1.01	12.9 ± 2.16	9.77E+03	0.61 ± 0.10	4.2 ± 0.71	0.7	2.32 ± 1.01
8	8	I	22.4 ± 2.04	1.02E+04	0.30 ± 0.03	2.1 ± 0.21	0.6	2.22 ± 0.67	16.2 ± 2.04	7.42E+03	0.37 ± 0.03	2.6 ± 0.23	0.5	2.40 ± 0.67
9	8	J	12.1 ± 1.08	1.24E+04	0.68 ± 0.07	4.7 ± 0.49	0.7	4.09 ± 0.82	17.4 ± 1.08	6.44E+03	0.30 ± 0.02	2.1 ± 0.14	0.5	3.21 ± 0.82
10	8	K	26.4 ± 2.21	1.17E+04	0.29 ± 0.03	2.0 ± 0.22	0.7	3.45 ± 1.55	27.4 ± 2.21	9.79E+03	0.29 ± 0.04	2.0 ± 0.28	0.7	2.51 ± 1.55
11	8	L	17.5 ± 1.93	1.25E+04	0.47 ± 0.06	3.3 ± 0.43	0.7	2.19 ± 0.42	14.8 ± 1.93	8.64E+03	0.47 ± 0.04	3.3 ± 0.28	0.6	1.57 ± 0.42
12	8	M	17.1 ± 1.15	1.24E+04	0.48 ± 0.04	3.3 ± 0.26	0.7	2.56 ± 0.66	6.7 ± 1.15	1.11E+04	1.34 ± 0.09	9.3 ± 0.63	0.8	3.26 ± 0.66
13	8	N	14.3 ± 0.47	1.79E+04	0.82 ± 0.03	5.7 ± 0.21	1.1	2.71 ± 0.42	10.4 ± 0.47	1.32E+04	1.03 ± 0.06	7.1 ± 0.45	1.0	2.24 ± 0.42
14	9	A	8.6 ± 0.13	8.33E+03	0.66 ± 0.01	4.7 ± 0.08	0.5	3.18 ± 0.33	9.4 ± 0.13	8.50E+03	0.62 ± 0.07	4.4 ± 0.48	0.5	3.18 ± 0.33
15	9	B	14.9 ± 0.72	1.36E+04	0.62 ± 0.03	4.5 ± 0.23	0.9	3.96 ± 0.38	14.5 ± 0.72	9.15E+03	0.43 ± 0.04	3.1 ± 0.31	0.6	4.34 ± 0.38
16	9	C	13.5 ± 1.13	1.23E+04	0.62 ± 0.05	4.5 ± 0.37	0.8	2.42 ± 0.15	7.0 ± 1.13	1.39E+04	1.37 ± 0.07	9.8 ± 0.53	0.9	3.63 ± 0.15
17	9	D	9.5 ± 0.44	1.40E+04	1.01 ± 0.05	7.2 ± 0.35	0.8	4.56 ± 0.52	17.0 ± 0.44	9.11E+03	0.37 ± 0.02	2.6 ± 0.13	0.6	3.22 ± 0.52
18	9	E	15.6 ± 2.06	1.62E+04	0.71 ± 0.09	5.1 ± 0.62	1.0	2.47 ± 0.56	12.7 ± 2.06	1.25E+04	0.67 ± 0.10	4.8 ± 0.74	0.8	2.60 ± 0.56
19	9	F	16.1 ± 0.59	1.22E+04	0.52 ± 0.02	3.7 ± 0.14	0.8	2.15 ± 0.11	10.7 ± 0.59	1.32E+04	0.84 ± 0.11	6.0 ± 0.76	0.8	2.71 ± 0.11
20	9	G	13.3 ± 1.85	1.54E+04	0.79 ± 0.12	5.6 ± 0.88	1.0	4.30 ± 0.24	9.9 ± 1.85	1.19E+04	0.82 ± 0.08	5.9 ± 0.59	0.8	4.24 ± 0.24

*The average error in PSC for both the INITIAL and FINAL states was in the range 7 10^{-6}-1.1 10^{-5} nmol/m².

B: (Non-leaky → non-leaky) GUVs

			INITIAL state						FINAL state					
	day	GUV	$\langle N\rangle$	average intensity ⟨cps⟩	$\langle \phi\rangle$	$\langle N(\mathrm{m.u.})\rangle$	PSC* nmol/m²	$\langle D\rangle$ µm²/s	$\langle N\rangle$	average intensity ⟨cps⟩	$\langle \phi\rangle$	$\langle N(\mathrm{m.u.})\rangle$	PSC* nmol/m²	$\langle D\rangle$ µm²/s
1	1	B	26.9 ± 3.85	1.69E+04	0.45 ± 0.08	3.2 ± 0.59	1.1	2.14 ± 1.54	13.3 ± 1.58	3.53E+04	1.88 ± 0.34	13.5 ± 2.41	2.3	2.42 ± 0.69
2	1	E	19.9 ± 1.97	1.60E+04	0.57 ± 0.07	4.1 ± 0.50	1.0	1.54 ± 0.13	10.2 ± 1.25	3.35E+04	2.32 ± 0.35	16.5 ± 2.49	2.2	1.96 ± 0.21
3	1	F	21.5 ± 4.54	5.18E+03	0.17 ± 0.06	1.2 ± 0.40	0.3	1.79 ± 2.32	13.2 ± 2.45	7.00E+03	0.38 ± 0.09	2.7 ± 0.66	0.5	3.70 ± 0.45
4	1	G	12.5 ± 3.33	1.44E+04	0.82 ± 0.18	5.9 ± 1.25	0.9	3.13 ± 0.66	14.2 ± 1.10	1.68E+04	0.84 ± 0.08	6.0 ± 0.59	1.1	2.25 ± 0.20
5	2	A	39.0 ± 13.37	1.49E+04	0.29 ± 0.09	2.0 ± 0.68	1.0	1.55 ± 0.90	6.2 ± 0.70	1.57E+04	1.91 ± 0.23	13.6 ± 1.67	1.1	1.76 ± 0.30
6	2	C	24.1 ± 3.30	1.40E+04	0.44 ± 0.05	3.1 ± 0.36	1.0	2.60 ± 0.55	4.6 ± 0.49	1.46E+04	2.37 ± 0.34	16.9 ± 2.41	1.0	2.01 ± 0.51
7	2	F	26.6 ± 1.70	3.13E+04	0.88 ± 0.06	6.3 ± 0.41	2.2	3.40 ± 1.24	11.5 ± 0.11	2.62E+04	1.71 ± 0.02	12.2 ± 0.12	1.8	1.87 ± 0.34
8	2	G	32.9 ± 2.81	5.71E+04	1.30 ± 0.11	9.3 ± 0.81	4.0	2.48 ± 0.48	12.8 ± 0.62	4.62E+04	2.71 ± 0.15	19.4 ± 0.12	3.2	1.24 ± 0.11
9	3	A	24.3 ± 3.28	1.70E+04	0.56 ± 0.08	4.0 ± 0.57	1.3	2.68 ± 1.20	12.5 ± 1.46	1.43E+04	0.92 ± 0.10	6.6 ± 1.08	1.1	2.08 ± 0.46
10	3	F	23.1 ± 3.23	1.28E+04	0.44 ± 0.08	3.2 ± 0.57	1.0	3.13 ± 1.20	11.8 ± 1.46	8.94E+03	0.61 ± 0.10	4.4 ± 0.70	0.7	2.15 ± 0.46
11	3	G	15.4 ± 1.59	3.21E+04	1.68 ± 0.19	12.0 ± 1.33	2.4	1.96 ± 0.34	7.6 ± 0.19	2.71E+04	2.86 ± 0.07	20.4 ± 0.70	2.0	2.17 ± 0.23
12	4	E	34.1 ± 2.99	2.82E+04	0.57 ± 0.05	4.1 ± 0.37	1.8	2.42 ± 0.89	13.9 ± 1.29	3.15E+04	1.57 ± 0.16	11.2 ± 0.52	2.0	2.42 ± 0.45
13	4	F	15.1 ± 1.53	5.03E+04	2.31 ± 0.24	16.5 ± 1.71	3.2	2.52 ± 0.07	13.5 ± 0.21	3.58E+04	1.84 ± 0.03	13.1 ± 1.15	2.3	1.13 ± 0.15
14	4	G	21.8 ± 3.23	1.98E+04	0.63 ± 0.09	4.5 ± 0.62	1.3	3.56 ± 0.97	14.5 ± 0.68	2.22E+04	1.06 ± 0.05	7.6 ± 0.22	1.4	2.79 ± 0.35
15	4	H	15.5 ± 0.32	2.91E+04	1.30 ± 0.03	9.3 ± 0.19	1.9	2.57 ± 0.20	13.0 ± 0.26	2.86E+04	1.52 ± 0.03	10.9 ± 0.37	1.8	2.04 ± 0.18
16	5	B	27.7 ± 2.61	9.35E+03	0.25 ± 0.13	1.8 ± 0.93	0.6	2.85 ± 0.43	8.9 ± 2.39	1.29E+04	1.07 ± 0.11	7.6 ± 0.23	0.9	3.00 ± 0.85
17	5	C	28.4 ± 9.08	7.87E+03	0.21 ± 0.10	1.5 ± 0.70	0.5	2.23 ± 0.42	7.8 ± 2.17	9.81E+03	0.93 ± 0.22	6.6 ± 0.80	0.7	2.74 ± 0.96
18	5	D	21.6 ± 1.23	8.98E+03	0.31 ± 0.03	2.2 ± 0.18	0.6	2.43 ± 0.99	4.9 ± 0.56	9.80E+03	1.48 ± 0.17	10.6 ± 1.60	0.7	3.13 ± 0.29
19	5	G	19.4 ± 1.0	2.44E+04	0.93 ± 0.05	6.7 ± 0.37	1.7	3.19 ± 0.51	11.7 ± 0.44	1.46E+04	0.93 ± 0.04	6.6 ± 1.23	1.0	2.45 ± 0.39
20	5	H	25.3 ± 3.96	1.52E+04	0.44 ± 0.08	3.2 ± 0.55	1.0	2.57 ± 1.37	8.8 ± 3.62	1.32E+04	1.12 ± 0.72	8.0 ± 0.27	0.9	2.27 ± 1.55
21	5	J	12.8 ± 1.03	1.32E+04	0.76 ± 0.07	5.5 ± 0.48	0.9	2.37 ± 0.30	8.7 ± 0.35	1.29E+04	1.09 ± 0.05	7.8 ± 5.18	0.9	2.67 ± 0.42
22	5	K	17.7 ± 2.09	2.06E+04	0.86 ± 0.12	6.1 ± 0.83	1.4	4.11 ± 2.20	6.1 ± 1.19	1.25E+04	1.51 ± 0.23	10.8 ± 0.35	0.9	4.13 ± 0.45
23	6	E	11.1 ± 0.67	1.34E+04	0.82 ± 0.05	5.8 ± 0.34	0.8	4.12 ± 0.40	6.3 ± 0.89	7.49E+03	0.80 ± 0.11	5.7 ± 1.65	0.5	3.35 ± 0.65
24	6	G	11.2 ± 0.16	1.54E+04	0.92 ± 0.01	6.6 ± 0.09	1.0	3.32 ± 0.17	8.1 ± 1.08	8.36E+03	0.70 ± 0.09	5.0 ± 0.75	0.5	2.27 ± 0.09
25	7	D	60.6 ± 5.09	2.82E+04	0.38 ± 0.03	2.7 ± 0.24	2.1	1.01 ± 0.29	13.3 ± 1.16	2.53E+04	1.54 ± 0.14	11.0 ± 0.62	1.9	1.68 ± 0.12
26	7	I	27.6 ± 1.06	3.95E+04	1.16 ± 0.05	8.3 ± 0.33	3.0	1.01 ± 0.09	134.0 ± 0.29	2.68E+04	1.55 ± 0.03	11.1 ± 1.02	2.0	0.40 ± 0.04
27	7	J	35.5 ± 3.85	2.61E+04	0.59 ± 0.06	4.2 ± 0.45	2.0	0.75 ± 0.13	15.2 ± 1.26	3.04E+04	1.62 ± 0.16	11.5 ± 0.24	2.3	0.72 ± 0.13

*The average error in PSC for both the INITIAL and FINAL states was in the range 1.72 10^{-5}-0.03 nmol/m².

C: (Non-Leaky → leaky) GUVs

			INITIAL state						FINAL state					
	day	GUV	$\langle N\rangle$	average intensity ⟨cps⟩	$\langle \phi\rangle$	$\langle N(\mathrm{m.u.})\rangle$	PSC* nmol/m²	$\langle D\rangle$ µm²/s	$\langle N\rangle$	average intensity ⟨cps⟩	$\langle \phi\rangle$	$\langle N(\mathrm{m.u.})\rangle$	PSC* nmol/m²	$\langle D\rangle$ µm²/s
1	1	C	18.0 ± 2.36	8.67E+03	0.34 ± 0.05	2.4 ± 0.38	0.6	1.48 ± 0.65	8.8 ± 2.10	1.67E+04	1.35 ± 0.71	9.7 ± 5.08	1.1	2.61 ± 0.23
2	1	D	11.3 ± 1.03	9.18E+03	0.58 ± 0.06	4.1 ± 0.40	0.6	2.94 ± 0.66	11.4 ± 0.80	1.47E+04	0.92 ± 0.07	6.6 ± 0.50	1.0	3.86 ± 0.25
3	1	H	16.3 ± 1.15	3.04E+04	1.32 ± 0.10	9.4 ± 0.69	2.0	2.02 ± 0.22	256.0 ± 1.11	5.03E+04	1.37 ± 0.06	9.8 ± 0.44	3.3	1.24 ± 0.20
4	1	I	13.8 ± 1.18	2.55E+04	1.31 ± 0.11	9.4 ± 0.79	1.7	3.20 ± 0.27	24.2 ± 2.03	5.34E+04	1.57 ± 0.13	11.2 ± 0.95	3.5	3.08 ± 0.16
5	2	B	34.8 ± 4.12	2.40E+04	0.52 ± 0.07	3.7 ± 0.49	1.7	1.04 ± 0.23	15.1 ± 0.95	3.19E+04	1.58 ± 0.02	11.3 ± 0.13	2.2	1.42 ± 0.25
6	2	H	22.9 ± 2.76	2.52E+04	0.82 ± 0.10	5.9 ± 0.69	1.7	3.40 ± 1.00	17.4 ± 0.58	2.48E+04	1.07 ± 0.03	7.6 ± 0.24	1.7	2.62 ± 0.12
7	3	B	27.9 ± 5.15	1.72E+04	0.50 ± 0.16	3.5 ± 1.13	1.3	2.17 ± 0.36	11.7 ± 0.68	1.63E+04	1.13 ± 0.08	8.0 ± 0.55	1.2	3.49 ± 0.37
8	4	A	32.9 ± 3.27	1.83E+04	0.39 ± 0.04	2.8 ± 0.28	1.2	1.52 ± 0.52	26.5 ± 3.24	3.23E+04	0.85 ± 0.13	6.1 ± 0.94	2.1	1.64 ± 0.15
9	4	B	20.7 ±2.61	2.77E+04	0.93 ± 0.12	6.6 ± 0.87	1.8	3.55 ± 0.43	18.8 ± 2.39	2.08E+04	0.77 ± 0.11	5.5 ± 0.75	1.3	3.12 ± 0.85
10	4	D	25.7 ± 1.60	2.41E+04	0.65 ± 0.04	4.7 ± 0.26	1.5	2.37 ± 0.22	15.8 ± 1.94	3.33E+04	1.47 ± 0.17	10.5 ± 1.21	2.1	2.33 ± 0.62
11	4	I	17.7 ±0.31	1.67E+04	0.65 ± 0.01	4.7 ± 0.08	1.1	3.04 ± 0.41	32.8 ± 2.73	3.34E+04	0.71 ± 0.07	5.0 ± 0.49	2.1	2.34 ± 0.71
12	4	J	9.7 ±0.04	2.56E+04	1.83 ± 0.01	13.1 ± 0.06	1.6	2.06 ± 0.06	14.7 ± 0.83	4.00E+04	1.89 ± 0.12	13.5 ± 0.83	2.6	1.68 ± 0.29
13	5	E	15.3 ± 2.01	1.59E+04	0.77 ± 0.11	5.5 ± 0.77	1.1	4.11 ± 0.80	10.6 ± 0.61	1.45E+04	1.02 ± 0.06	7.3 ± 0.44	1.0	4.28 ± 0.95
14	5	F	16.8 ± 1.60	2.42E+04	1.06 ± 0.09	7.6 ± 0.67	1.7	3.09 ± 0.36	13.4 ± 1.30	1.36E+04	0.75 ± 0.08	5.4 ± 0.57	0.9	3.43 ± 0.05
15	6	D	16.1 ± 1.83	1.91E+04	0.80 ± 0.10	5.7 ± 0.68	1.2	2.83 ± 1.11	10.6 ± 0.73	1.35E+04	0.86 ± 0.06	6.1 ± 0.46	0.8	2.61 ± 0.51
16	6	F	6.8 ± 0.38	6.26E+03	0.62 ± 0.03	4.4 ± 0.25	0.4	3.56 ± 0.99	10.9 ± 0.93	5.14E+03	0.32 ± 0.03	2.3 ± 0.22	0.3	4.65 ± 1.20
17	6	H	14.4 ± 0.74	2.13E+04	1.00 ± 0.05	7.1 ± 0.37	1.3	3.38 ± 0.47	12.4 ± 0.49	1.25E+04	0.68 ± 0.03	4.8 ± 0.20	0.8	3.48 ± 0.35
18	6	I	13.1 ± 0.70	1.77E+04	0.91 ± 0.05	6.5 ± 0.39	1.1	3.07 ± 0.22	11.4 ± 0.26	1.27E+04	0.75 ± 0.02	5.4 ± 0.14	0.8	2.53 ± 0.55
19	7	B	112.8 ± 20.3	3.28E+04	0.24 ± 0.06	1.7 ± 0.40	2.5	0.55 ± 1.17	22.4 ± 2.27	5.54E+04	2.00 ± 0.21	14.3 ± 1.49	4.1	0.86 ± 0.16
20	7	C	39.2 ± 7.42	16382	0.25 ± 0.04	1.8 ± 0.28	0.9	3.02 ± 0.16	23.1 ± 2.01	13467	0.35 ± 0.03	2.5 ± 0.18	0.7	10.9 ± 0.16

*The average error in PSC for both the INITIAL and FINAL states was in the range 1.33 10^{-5}-2.6 10^{-5} nmol/m².

References

(1) Steringer, J. P.; Lange, S.; Čujová, S.; Šachl, R.; Poojari, C.; Lolicato, F.; Beutel, O.; Müller, H. M.; Unger, S.; Coskun, Ü.; Honigmann, A.; Vattulainen, I.; Hof, M.; Freund, C.; Nickel, W. Key Steps in Unconventional Secretion of Fibroblast Growth Factor 2 Reconstituted with Purified Components. *Elife* **2017**, *6*, 1–36. https://doi.org/10.7554/eLife.28985.
